# Efficacy and Safety of S-1 Compared With Docetaxel in Elderly Patients With Advanced NSCLC Previously Treated With Platinum-Based Chemotherapy: A Subgroup Analysis of the EAST-LC Trial

**DOI:** 10.1016/j.jtocrr.2021.100142

**Published:** 2021-01-07

**Authors:** James Chih-Hsin Yang, Tony S.K. Mok, Shun Lu, Kazuhiko Nakagawa, Nobuyuki Yamamoto, Yuan-Kai Shi, Li Zhang, Ross A. Soo, Satoshi Morita, Tomohide Tamura

**Affiliations:** a Department of Oncology, National Taiwan University Hospital and National Taiwan University Cancer Center, Taipei, Republic of China; b Department of Clinical Oncology, State Key Laboratory of Translational Oncology, Chinese University of Hong Kong, Shatin, Hong Kong Special Administrative Region, People’s Republic of China; c Shanghai Lung Cancer Center, Shanghai Chest Hospital, Shanghai Jiao Tong University, Shanghai, People’s Republic of China; d Department of Medical Oncology, Kindai University Faculty of Medicine, Osaka-sayama, Japan; e Internal Medicine III, Wakayama Medical University, Wakayama, Japan; f Department of Medical Oncology, National Cancer Center/National Clinical Research Centre for Cancer/Cancer Hospital, Chinese Academy of Medical Sciences & Peking Union Medical College, Beijing, People’s Republic of China; g Department of Medical Oncology, Sun Yat-Sen University Cancer Center, Guangzhou, People’s Republic of China; h Department of Hematology-Oncology, National University Hospital, Singapore; i Department of Biomedical Statistics and Bioinformatics, Kyoto University Graduate School of Medicine, Kyoto, Japan; j Thoracic Center, St Luke’s International Hospital, Tokyo, Japan

**Keywords:** Non–small cell lung cancer, S-1, Elderly, Platinum-based chemotherapy, Phase 3 clinical trial

## Abstract

**Introduction:**

Despite recent advances in NSCLC treatment, specific data on the elderly population remain limited. In this post hoc subgroup analysis of the East Asia S-1 Trial in Lung Cancer (EAST-LC) trial, we compared S-1 and docetaxel (DTX) in patients aged 70 years old and above with pretreated advanced NSCLC.

**Methods:**

Patients were randomly assigned (1:1) to receive S-1 (orally, twice daily on d 1–28 of a 6-wk cycle) or DTX (intravenously, on d 1 of a 3-wk cycle). The initial S-1 dose was 80, 100, or 120 mg/day on the basis of body surface area, and the DTX doses were 60 mg/m^2^ (Japan) or 75 mg/m^2^ (outside Japan). The primary end point was overall survival, and secondary end points included progression-free survival, response rate, quality of life (QOL) using the European Organisation for Research and Treatment of Cancer Quality of Life Questionnaire Core-30, and safety.

**Results:**

Among 189 patients aged 70 years and above assessed as the full analysis set, baseline characteristics were generally similar between treatment arms. The median overall survival was 14.7 (S-1) versus 12.1 months (DTX); the hazard ratio was equal to 0.76, with a 95% confidence interval (CI) of 0.54–1.07. The median progression-free survival was similar in both arms (both 4.1 mo, hazard ratio = 0.84, 95% CI: 0.60–1.18); and the response rate was 12.9% (S-1) and 14.0% (DTX). The adjusted mean QOL score difference (S-1–DTX until wk 48) was 7.41 (95% CI: 0.37–14.46). Safety profiles were generally consistent with those of the overall EAST-LC population.

**Conclusions:**

S-1 revealed comparable efficacy, safety, and QOL versus DTX in pretreated elderly patients with advanced NSCLC. Results were consistent with the overall EAST-LC data.

## Introduction

According to global estimates, the highest rates of morbidity and mortality in patients with cancer are associated with lung cancer.^1^^,^^2^ In Eastern Asia, lung cancer affects 47.2 men and 21.9 women per 100,000 and is the leading cause of cancer death in men.^2^ During the past two decades, the incidence of lung cancer in East Asian countries has remained high,^3^ and the burden is expected to increase as the population ages.^4^ Approximately 84% of all lung cancer cases are NSCLC,^5^ and this form of cancer is common in the elderly.^6^ An analysis of the Surveillance, Epidemiology, and End Results database indicated that 47% of patients with NSCLC in the United States were aged 70 years old and above, and 14% were at least 80 years.^7^ The more vulnerable clinical profile of elderly patients with NSCLC, owing to poor organ function reserve, polypharmacy, or comorbidities, may make it difficult to administer treatment with the same intensity as that used for younger patients.^8^ In elderly patients with NSCLC receiving chemotherapy, pretreatment quality of life (QOL) was found to be a prognostic factor for survival outcomes.^9^ In addition, treatment decisions for elderly patients are complex and should primarily focus on maintenance or improvement of QOL and functional status.^10^

Current treatment recommendations suggest the use of targeted therapy as the first-line treatment in elderly patients with advanced NSCLC if there are oncogenic driver mutations, whereas single-agent chemotherapy or carboplatin-based doublet chemotherapy should be initiated in patients without driver mutations or as systemic therapy after completion of targeted therapy.^10^, ^11^, ^12^, ^13^ In addition, data from recent studies have revealed that monotherapy with the immune checkpoint inhibitor (ICI) pembrolizumab,^14^^,^^15^ or combination therapy with pembrolizumab plus chemotherapy,^16^^,^^17^ or atezolizumab plus chemotherapy^18^^,^^19^ produces superior survival benefits over platinum-based chemotherapy alone; as a result, these therapies have been considered as the standard first-line treatments for NSCLC without driver mutations, regardless of age. For second- or later-line treatment in patients with advanced NSCLC, single-agent chemotherapy, immunotherapies, and combination docetaxel (DTX) plus ramucirumab (RAM) are also recommended as systemic therapy, regardless of age.^10^, ^11^, ^12^, ^13^ In general, DTX plus RAM and ICIs exhibit superior survival benefits over DTX alone,^20^, ^21^, ^22^, ^23^, ^24^, ^25^, but DTX monotherapy remains widely used as second-line therapy in clinical practice.^26^

S-1 is a formulation composed of tegafur (a prodrug of 5-fluorouracil [5-FU]) with the modulators gimeracil (which reversibly inhibits the 5-FU catabolic enzyme dihydropyrimidine dehydrogenase to maintain the concentration of 5-FU, thus facilitating cytotoxicity) and oteracil potassium (which selectively inhibits phosphorylation of 5-FU by orotate phosphoribosyltransferase in the gastrointestinal tract, thereby decreasing gastrointestinal toxicity) at a molar ratio of 1-to-0.4-to-1.^27^^,^^28^ The East Asia S-1 Trial in Lung Cancer (EAST-LC) was a randomized, controlled, phase 3 trial conducted in Asia, comparing S-1 with DTX in patients with previously treated advanced NSCLC.^29^ The results of the EAST-LC trial established the noninferiority of S-1 to DTX for overall survival (OS) (hazard ratio [HR] = 0.95, 95% confidence interval [CI]: 0.83–1.07) and also exhibited a favorable QOL profile (assessed using the European Organisation for Research and Treatment of Cancer Quality of Life Questionnaire Core-30 [EORTC QLQ-C30]). On the basis of these data, S-1 is one of the recommended regimens for second- or later-line chemotherapy in patients with advanced NSCLC in the current Japanese guidelines^10^ and has also been recently approved for NSCLC in the Republic of China and South Korea.

Despite the many recent advances in NSCLC treatment, there is limited available information on efficacy and safety outcomes for previously treated elderly patients. Most subgroup analyses for the ICIs were stratified on the basis of age less than 65 and 65 years old and above,^30^ although one recent pooled analysis of pembrolizumab clinical trials reported that outcomes in patients aged 75 years and above were comparable with those observed in the overall populations in the individual studies.^31^ Previous single-arm phase 2 studies evaluating the efficacy and safety of S-1 in the first-line setting reported S-1 to be effective in elderly patients with manageable toxicity^32^, ^33^, ^34^; however, there is a lack of robust data for previously treated patients. In terms of administering chemotherapy in elderly patients with NSCLC, this remains controversial because elderly patients are often excluded from prospective clinical trials.^35^ Therefore, we conducted a post hoc subgroup analysis using data from the EAST-LC trial to assess the clinical outcomes associated with the use of S-1 or DTX in patients aged 70 years and above.

## Materials and Methods

### Trial Design and Patients

This post hoc analysis used data collected during the EAST-LC trial (JapicCTI-101155), a randomized, open-label, phase 3 noninferiority trial that was conducted at 84 medical centers in the People’s Republic of China (including Hong Kong), Japan, Singapore, and the Republic of China.^29^ The EAST-LC primary article has been published.^29^ In brief, patients with locally advanced or metastatic NSCLC (clinical stage IIIB or IV, with measurable or nonmeasurable lesions), were eligible for enrollment if they were at least 20 years of age, had an Eastern Cooperative Oncology Group performance status (ECOG PS) greater than or equal to 2, and had received one or two previous chemotherapy regimens (including a platinum-based regimen) or three previous regimens (including an EGFR tyrosine kinase inhibitor [EGFR TKI] such as gefitinib or erlotinib).

The trial was conducted in accordance with the Good Clinical Practice guidance set out by the International Conference on Harmonization, the ethical principles outlined in the Declaration of Helsinki, and all applicable national and international regulatory requirements. The protocol was approved by the institutional review board or independent ethics committee at each trial center. All patients provided written informed consent before enrollment in the trial.

### Treatment

Patients were randomly assigned to receive either S-1 or DTX. S-1 was administered orally in a 6-week cycle, given twice daily after meals on days 1 to 28. The initial dose for patients receiving S-1 was 80 mg/day, 100 mg/day, or 120 mg/day and was determined on the basis of body surface area. DTX was administered in a 3-week cycle, given intravenously on day 1. The DTX doses were 60 mg/m^2^ in Japan and 75 mg/m^2^ in the Republic of China, Singapore, and the P.R. China including Hong Kong. Patients received treatment until disease progression, unacceptable toxicity, or patient withdrawal.

### Outcomes and Assessments

For this analysis, we evaluated the following: (1) OS, defined as the time between random assignment and death from any cause; (2) progression-free survival (PFS), defined as the time between random assignment and the earliest event of either progression or death from any cause; (3) response rate (RR), defined as the proportion of patients with complete response or partial response as the best overall response; (4) posttrial treatment; (5) QOL; and (6) safety.

Tumor imaging (by computed tomography, magnetic resonance imaging, or radiograph of the chest, abdomen, and head) was conducted every 6 weeks until radiologic progression was confirmed. Tumor response was assessed in patients with measurable lesions according to the Response Evaluation Criteria in Solid Tumors (version 1.1). QOL assessments were performed every 6 weeks and at the end of therapy or patient withdrawal, using the EORTC QLQ-C30. The QOL instructions were administered before a clinic visit. For safety outcomes, adverse events (AEs) were recorded throughout the trial and classified using the Common Terminology Criteria for Adverse Events version 4.0.

### Statistical Analysis

For this post hoc analysis, the full analysis set (FAS) consisted of all randomized patients aged 70 years and above, except those with a major protocol deviation. The cutoff of 70 years was based on current practice guidelines.^12^^,^^13^ The safety analysis set consisted of all patients aged greater than or equal to 70 years who received at least one dose of the trial drug.

The full details of the overall statistical analysis have been reported previously.^29^ In this analysis, statistical calculations were performed using SAS version 9.4 (SAS Institute Inc., Cary, NC). Briefly, the OS and PFS rates were calculated using the Kaplan-Meier method; HRs were calculated using a Cox proportional hazards model, including treatment, performance status, number of previous chemotherapy regimens, EGFR TKI in previous treatments, EGFR mutation status, sex, histologic type, and smoking status as covariates. RR values and associated two-sided 95% CIs were calculated. QOL variables were summarized descriptively with mean and SE, and a linear mixed-effect model was used to analyze changes over time. The efficacy analysis and QOL assessments were based on the FAS, and the safety assessments were based on the safety analysis set.

## Results

### Patient Characteristics and Drug Delivery

**Figure 1:**
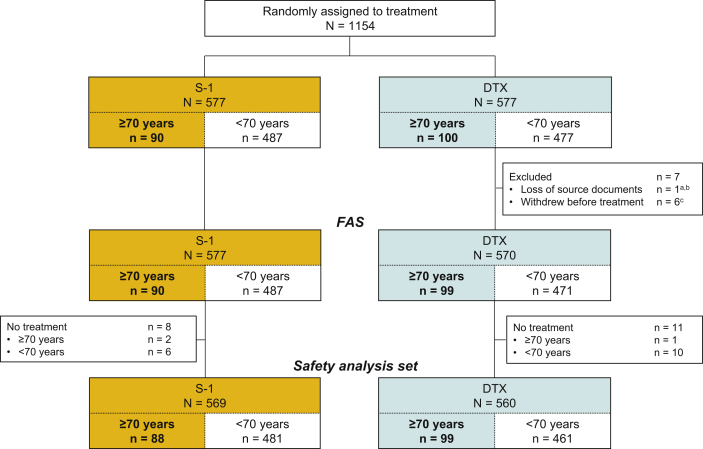
Patient disposition. Trial scheme outlining the flow of elderly (aged ≥70 y) patients and the resulting analysis sets. ^a^Included in the safety analysis set, but not in the FAS. ^b^≥70 years. ^c^All <70 years. DTX, docetaxel; FAS, full analysis set.

A total of 190 elderly patients (aged ≥70 y) were included in this analysis (16.5% of the overall EAST-LC population). The FAS included 90 patients in the S-1 arm and 99 patients in the DTX arm; the safety analysis set included 88 patients in the S-1 arm and 99 patients in the DTX arm. The full details, including reasons for exclusion, are illustrated in Figure 1.

The baseline characteristics of elderly patients are illustrated in Table 1. The median age was 73 years (range: 70–85) in the S-1 arm and 72 years (range: 70–82) in the DTX arm. There were no significant differences between arms in terms of baseline characteristics, with the exception of ECOG PS (*p* = 0.0132).

**Table 1:** Patient Demographic and Clinical Characteristics of Elderly (Aged ≥70 y) Patients at Baseline (FAS)

Characteristics	S-1 (N = 90)	DTX (N = 99)
Male sex, n (%)	60 (66.7)	67 (67.7)
Age, median (range)	73 (70–85)	72 (70–82)
Ethnicity, n (%)		
Japanese	82 (91.1)	83 (83.8)
Chinese	8 (8.9)	13 (13.1)
Taiwanese	0 (0)	3 (3.0)
ECOG performance status, n (%)		
0	44 (48.9)	31 (31.3)
1	44 (48.9)	64 (64.6)
2	2 (2.2)	4 (4.0)
Histologic diagnosis, n (%)		
Adenocarcinoma	57 (63.3)	71 (71.7)
Squamous cell carcinoma	26 (28.9)	23 (23.2)
Large-cell carcinoma	2 (2.2)	3 (3.0)
Other	5 (5.6)	2 (2.0)
No. of previous treatments, n (%)		
1	60 (66.7)	64 (64.6)
2	25 (27.8)	28 (28.3)
3	5 (5.6)	7 (7.1)
EGFR status, n (%)		
Wild-type	55 (61.1)	49 (49.5)
Mutant	21 (23.3)	24 (24.2)
Unknown	14 (15.6)	26 (26.3)
Previous EGFR TKI, n (%)		
No	74 (82.2)	75 (75.8)
Yes	16 (17.8)	24 (24.2)

DTX, docetaxel; ECOG, Eastern Cooperative Oncology Group; FAS, full analysis set; TKI, tyrosine kinase inhibitor.

The median duration of treatment was two 6-weekly cycles (range: 1–27) in the S-1 arm and four 3-weekly cycles (range: 1–16) in the DTX arm, and the relative dose intensities of S-1 and DTX were 84.7% and 93.3%, respectively. At the data cutoff date (November 20, 2015), all patients aged 70 years and above had discontinued trial drug treatment. The number of patients aged 70 years and above who had dose delay was 32 (36.4%) and 55 (55.6%) in the S-1 and DTX arms, respectively, and that of dose reduction was 24 (27.3%) and 30 (30.3%) in the S-1 and DTX arms, respectively. Disease progression was the most common reason for treatment discontinuation in both groups (63.6% in the S-1 arm and 51.5% in the DTX arm), followed by AEs (13.6% in the S-1 arm and 24.2% in the DTX arm) (Table 2).

**Table 2:** Reasons for Discontinuation (Safety Analysis Set)

Reason	S-1 (N = 88)	DTX (N = 99)	OR (95% CI)
Progressive disease	56 (63.6)	51 (51.5)	1.65 (0.92–2.96)
AE^a^	12 (13.6)	24 (24.2)	0.49 (0.23–1.06)
Patient refusal	12 (13.6)	9 (9.1)	1.58 (0.63–4.06)
Other^b^	8 (9.1)	15 (15.2)	0.56 (0.23–1.39)

*Note:* Values are given in n (%) unless indicated otherwise.

AE, adverse event; CI, confidence interval; DTX, docetaxel.

a Included grade greater than or equal to 3 peripheral motor or sensory neuropathy; grade greater than or equal to 2 pneumonitis; grade 4 nonhematologic toxicity; or any other AE that would prevent continued trial treatment (investigator’s opinion).

b Included failure to start treatment within 14 days of randomization; necessity of additional DTX dose reduction; patient situation; ineligible (per protocol); or other reason (according to discontinuation criteria).

### Efficacy

**Figure 2:**
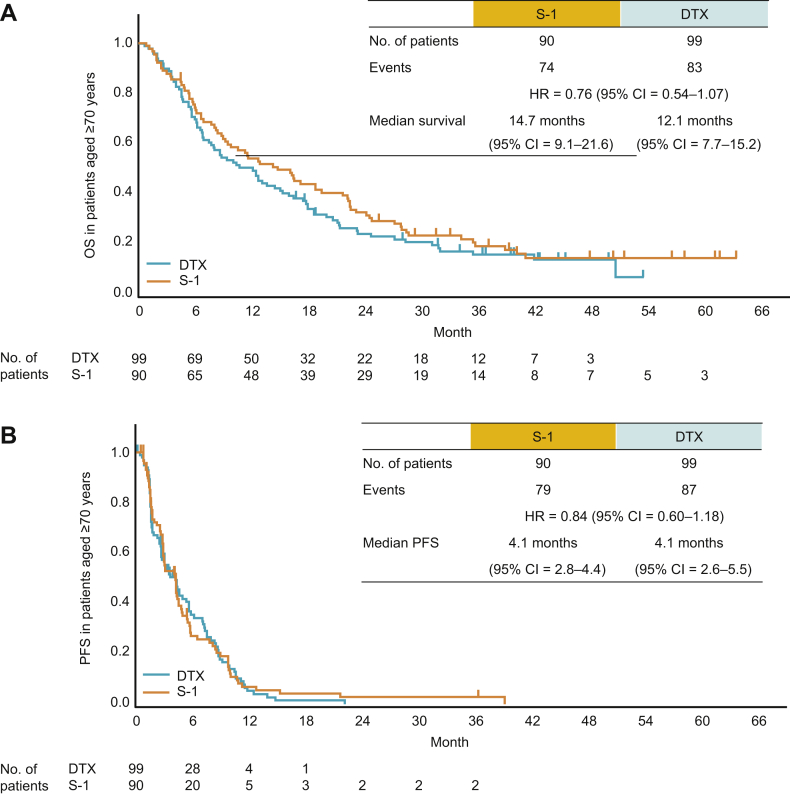
Kaplan-Meier graphical representations of survival in elderly (aged ≥70 y) patients. (*A*) OS. (*B*) PFS. Whiskers indicate censoring. CI, confidence interval; DTX, docetaxel; HR, hazard ratio; OS, overall survival: PFS, progression-free survival.

The median OS was 14.7 months for S-1 versus 12.1 months for DTX; HR 0.76 (95% CI: 0.54–1.07) (Fig. 2*A*). The median PFS durations were similar between the S-1 and DTX arms (4.1 and 4.1 mo, respectively, HR = 0.84, 95% CI: 0.60–1.18) (Fig. 2*B*). The OS data in 56 patients aged 75 years and above were comparable to those in patients aged 70 years and above (see Fig., Supplementary Data 1, showing the Kaplan-Meier OS estimates for patients aged ≥75 y in each treatment arm). The RR in patients with measurable lesions was 12.9% (n = 9 of 70) in the S-1 arm and 14.0% (n = 12 of 86) in the DTX arm (Supplementary Data 2, showing the best overall responses). In the S-1 and DTX arms, posttrial treatment was administered in 65.6% and 68.7% of patients, respectively, and a subsequent EGFR TKI was administered in 23.3% and 24.2% of patients, respectively (Supplementary Data 3, showing a breakdown of posttrial treatment by agent).

### Quality of Life

**Figure 3:**
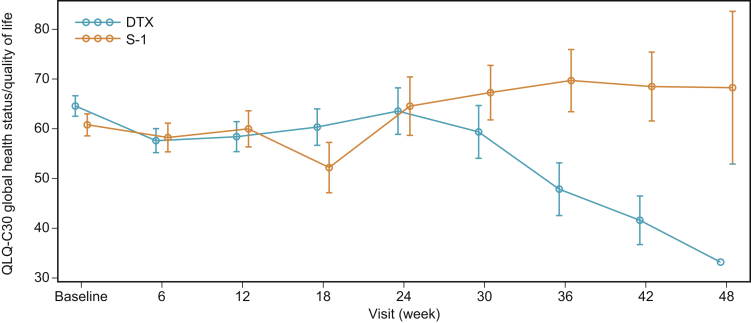
Mean (SE) EORTC QLQ-C30 global health status for the elderly (aged ≥70 y) patients. The adjusted mean score difference in EORTC QLQ-C30 between the S-1 and DTX arms until week 48 was 7.4 (95% CI: 0.4–14.5, *p* = 0.0393). CI, confidence interval; DTX, docetaxel; EORTC QLQ-C30, European Organisation for Research and Treatment of Cancer Quality of Life Questionnaire Core-30.

Changes in EORTC QLQ-C30 global health status over time up to 48 weeks are illustrated in Figure 3. The adjusted mean score difference (S-1–DTX until wk 48) on the basis of the linear model was 7.41 (95% CI: 0.37–14.46), which was comparable with that of the overall EAST-LC population.^29^

### Adverse Events

A summary of treatment-related AEs occurring in patients receiving S-1 or DTX is provided in Table 3. In general, the treatment-related AE profiles in each treatment arm in elderly patients were similar to those of the overall EAST-LC population, although some differences were observed. In patients aged 70 years and above receiving S-1, rates of decreased appetite (61.4%), diarrhea (47.7%), and stomatitis (38.6%) of any grade were greater than 10% higher than rates in the overall population (50.4%, 35.9%, and 23.4%, respectively^29^). Similarly, in the DTX arm, neutropenia (63.6%) and leukocytopenia (40.4%) grade 3 or worse were greater than 10%, higher than the rates reported in the overall population (47.7% and 29.1%, respectively^29^).

**Table 3:** Treatment-Related AEs Occurring in Greater Than or Equal to 10% of Patients in Either Arm (Safety Analysis Set)

Treatment-Related AEs	S-1 (N = 88)		DTX (N = 99)	
	Any Grade	Grade ≥3	Any Grade	Grade ≥3^a^
Hematologic				
Anemia	14 (15.9)	2 (2.3)	12 (12.1)	2 (2.0)
Neutropenia	10 (11.4)	5 (5.7)	66 (66.7)	63 (63.6)
Thrombocytopenia	8 (9.1)	2 (2.3)	4 (4.0)	0 (0)
Leukocytopenia	6 (6.8)	2 (2.3)	47 (47.5)	40 (40.4)
Febrile neutropenia	1 (1.1)	1 (1.1)	19 (19.2)	19 (19.2)
Nonhematologic				
Decreased appetite	54 (61.4)	12 (13.6)	46 (46.5)	5 (5.1)
Diarrhea	42 (47.7)	11 (12.5)	15 (15.2)	2 (2.0)
Skin hyperpigmentation	35 (39.8)	0 (0)	3 (3.0)	0 (0)
Stomatitis	34 (38.6)	4 (4.5)	15 (15.2)	1 (1.0)
Nausea	31 (35.2)	4 (4.5)	27 (27.3)	0 (0)
Fatigue	23 (26.1)	3 (3.4)	18 (18.2)	1 (1.0)
Malaise	18 (20.5)	0 (0)	27 (27.3)	1 (1.0)
Vomiting	15 (17.0)	2 (2.3)	8 (8.1)	1 (1.0)
Rash maculopapular	15 (17.0)	0 (0)	6 (6.1)	0 (0)
Constipation	13 (14.8)	0 (0)	19 (19.2)	0 (0)
Pyrexia	13 (14.8)	0 (0)	12 (12.1)	0 (0)
Lacrimation increased	11 (12.5)	2 (2.3)	3 (3.0)	0 (0)
Dry skin	10 (11.4)	0 (0)	6 (6.1)	0 (0)
Weight loss	10 (11.4)	1 (1.1)	9 (9.1)	0 (0)
Dysgeusia	10 (11.4)	0 (0)	14 (14.1)	0 (0)
Palmar-plantar erythrodysesthesia	9 (10.2)	1 (1.1)	1 (1.0)	0 (0)
Peripheral sensory neuropathy	5 (5.7)	0 (0)	13 (13.1)	2 (2.0)
Peripheral edema	3 (3.4)	0 (0)	21 (21.2)	1 (1.0)
Alopecia	1 (1.1)	0 (0)	49 (49.5)	0 (0)

*Note:* Values are given in n (%).

AE, adverse event; DTX, docetaxel

a One treatment-related death was observed in the DTX arm (ileus).

## Discussion

Here, we report the results of a post hoc subgroup analysis of elderly patients (aged ≥70 y) enrolled in the EAST-LC trial and confirm that the efficacy, safety, and QOL exhibited by S-1 were comparable with those of DTX. These data are also similar to that of the overall EAST-LC population,^29^ and provide support for the use of S-1 in elderly patients with advanced or metastatic NSCLC who have previously progressed on platinum-based chemotherapy. To our knowledge, this analysis represents the most detailed evaluation of the safety and efficacy of S-1 for elderly patients with previously treated advanced NSCLC.

Of particular note, OS and QOL data associated with S-1 were more favorable compared with the DTX data, although the duration of PFS and RR were equivalent between treatment arms. Although it is well known that posttrial treatment and postprogression survival can positively impact OS regardless of PFS results,^36^^,^^37^ in our analysis, the proportion of patients who received posttrial treatment was similar in both the S-1 and DTX arms. Furthermore, the details of the posttrial treatments, including the rates of administration of molecularly targeted drugs such as EGFR TKIs, were also similar between treatment arms. However, we noted that withdrawal owing to AEs occurred more frequently in the DTX arm compared with the S-1 arm, and we can hypothesize that the influence of these AEs may have led to reduced tolerability and continuity in the posttrial treatments, resulting in shorter postprogression survival in the DTX arm compared with the S-1 arm. In a previously published phase 3 trial comparing second-line pemetrexed with DTX in patients with advanced NSCLC, a subgroup analysis of patients aged 70 years and above reported a longer OS duration in the pemetrexed arm versus DTX (HR = 0.86).^38^ Weiss et al.^38^ suggested that fewer toxicities in the pemetrexed arm, especially febrile neutropenia, may have been one reason for the improvement of OS comparing with DTX. On the basis of the frequency of febrile neutropenia in the current analysis being 19.2% in the DTX arm and 1.1% in the S-1 arm, we consider that febrile neutropenia may influence OS in elderly patients. Moreover, elderly patients with NSCLC with an ECOG PS of 2 have been reported to have a poorer prognosis compared with those with a PS of 0 to 1,^9^ which indicates that general pretreatment health status can also contribute to OS. Although our efficacy results might have been influenced by the difference in ECOG PS observed between the S-1 and DTX arms (*p* = 0.0132), the adjusted HR including ECOG PS as a covariate illustrated better survival for elderly patients in the S-1 arm versus DTX (HR = 0.76).

In this analysis, the QOL outcome up to week 48 (assessed using the EORTC QLQ-C30 global health status) corresponded with the data reported for the overall EAST-LC population.29 For elderly patients with advanced disease, the presence of comorbidities or organ dysfunction and the use of polypharmacy must be taken into consideration during clinical decision-making^8^; thus, stabilization and improvement of QOL and functional status may become a higher priority than the prolongation of OS in elderly patients.^39^ As a result, the QOL observed in elderly patients with NSCLC who received S-1 in our study is considered to be clinically relevant and meaningful.

In general, S-1 and DTX in elderly patients exhibited a similar safety profile to that observed in the overall EAST-LC population.^29^ The AEs that occurred more frequently in the elderly patients in our analysis were decreased appetite, diarrhea, and stomatitis in the S-1 arm, and neutropenia and leukocytopenia in the DTX arm; all of these toxicities were manageable. In the EAST-LC trial, the S-1 treatment schedule was daily administration on days 1 to 28, followed by a 2-week discontinuation period within a 6-week cycle.^29^ However, if the neutrophil count was 500/mm^3^ or higher but less than 1000/mm^3^, the platelet count was at least 50,000/mm^3^ but less than 75,000/mm^3^, or if grade 2 or worse diarrhea, lack of appetite, or oral mucositis occurred from day 15 to day 29 of treatment, the schedule could be changed to a 3-week cycle comprising S-1 administration on days 1 to 14, followed by a 1-week discontinuation period. Of the 88 patients who received S-1 (safety analysis set), 23 patients (26.7%) were switched to 2 weeks on and 1 week off cycle. In a randomized trial of S-1 for postsurgery adjuvant chemotherapy in patients with head and neck cancer comparing the 3-week and 6-week cycle schedules, the 3-week schedule was reported to be more feasible.^40^ We consider that this 3-week schedule may also be considered as an option for treating elderly patients in clinical practice, especially for those in whom gastrointestinal toxicity may be a concern.

Previous publications have reported on the use of other second-line treatment regimens for advanced NSCLC; these include DTX plus RAM, or ICIs (nivolumab, pembrolizumab, and atezolizumab), each of which has exhibited survival benefit compared with DTX in this indication.^20^, ^21^, ^22^, ^23^, ^24^, ^25^ In a subgroup analysis of patients aged 70 years and above from the phase 3 REVEL trial, the OS HR for DTX plus RAM versus DTX plus placebo was 1.07, with no observable benefits in the DTX plus RAM arm.^20^ Moreover, as concomitant use of RAM has been reported to increase hematotoxicity,^41^ its use in patients aged 70 years and above requires careful consideration. Regarding treatment outcomes with ICIs, results from phase 3 studies suggest that the use of ICI monotherapy may benefit both elderly patients and younger (<65 y) patients; the reported HRs for OS versus DTX were 1.85 (nivolumab, patients aged ≥75 y with a squamous disease),^23^ 0.90 (nivolumab, patients aged ≥75 y with a nonsquamous disease),^22^ 0.76 (pembrolizumab, ≥65 y),^24^ and 0.66 (atezolizumab, ≥65 y).^25^ In addition, a prospective phase 2 trial of nivolumab in previously treated patients with NSCLC aged greater than or equal to 70 years has also reported favorable efficacy and safety outcomes.^42^ However, because ICIs are increasingly used as first-line treatments, and the benefit of sequential ICI regimen remains unclear, it is more likely that other anticancer agents will be chosen as second- or later-line treatments. It is not possible to directly compare the outcomes observed with DTX plus RAM or ICIs with the data reported with S-1 in this analysis; this is because the available data were obtained from subgroup analyses, and there are notable variations in the patient selection criteria (particularly the age cutoffs) and trial methodologies. However, we consider that the illustrated efficacy, QOL, and safety in elderly patients in our analysis, which are consistent with the data from the overall EAST-LC population, indicate that S-1 may be considered as a potential anticancer option for second- or later-line treatment of elderly patients with advanced NSCLC.

This analysis has several limitations. Given that this is a post hoc analysis, the population size was relatively small and has limited the study power. As a result, imbalance in baseline characteristics such as ECOG PS may confound the survival outcomes. Second, the study population for this analysis could be highly selected owing to the nature of the participants enrolled in the original clinical trial; thus, the results may not be generalizable to the broader elderly population. Third, the influence of previous ICI treatment on the efficacy and safety of S-1 or DTX is unclear because ICIs were not generally available at the time when the trial was conducted. Therefore, it will be important to prospectively investigate the efficacy of S-1 in elderly patients in future studies. Finally, most of the patients in this analysis received a DTX dose of 60 mg/m^2^ (the standard dose in Japan) rather than 75 mg/m^2^ (the standard dose in Western populations). A previous study^29^ did not detect a statistically significant difference in PFS or OS according to these different DTX doses; however, the number of patients in the current study who received 75 mg/m^2^ was too low to allow any statistical comparisons to be conducted.

In conclusion, S-1 exhibited comparable efficacy, safety, and QOL to DTX in the second- or later-line treatment of elderly (aged ≥70 y) patients with previously treated NSCLC; these results were consistent with the results obtained for the overall EAST-LC population. These results could support the use of S-1 as a viable treatment option for elderly patients with NSCLC with advanced disease.
