# Supplementary material for: Efficacy and Safety of S-1 Compared With Docetaxel in Elderly Patients With Advanced NSCLC Previously Treated With Platinum-Based Chemotherapy: A Subgroup Analysis of the EAST-LC Trial
Source: JTO Clin Res Rep. 2021 Jan 7;2(3):100142. doi: 10.1016/j.jtocrr.2021.100142 (PMC8474214; doi:10.1016/j.jtocrr.2021.100142)
Supplement: Supplemental Data 1 [file mmc1.pptx]

## Slide 1
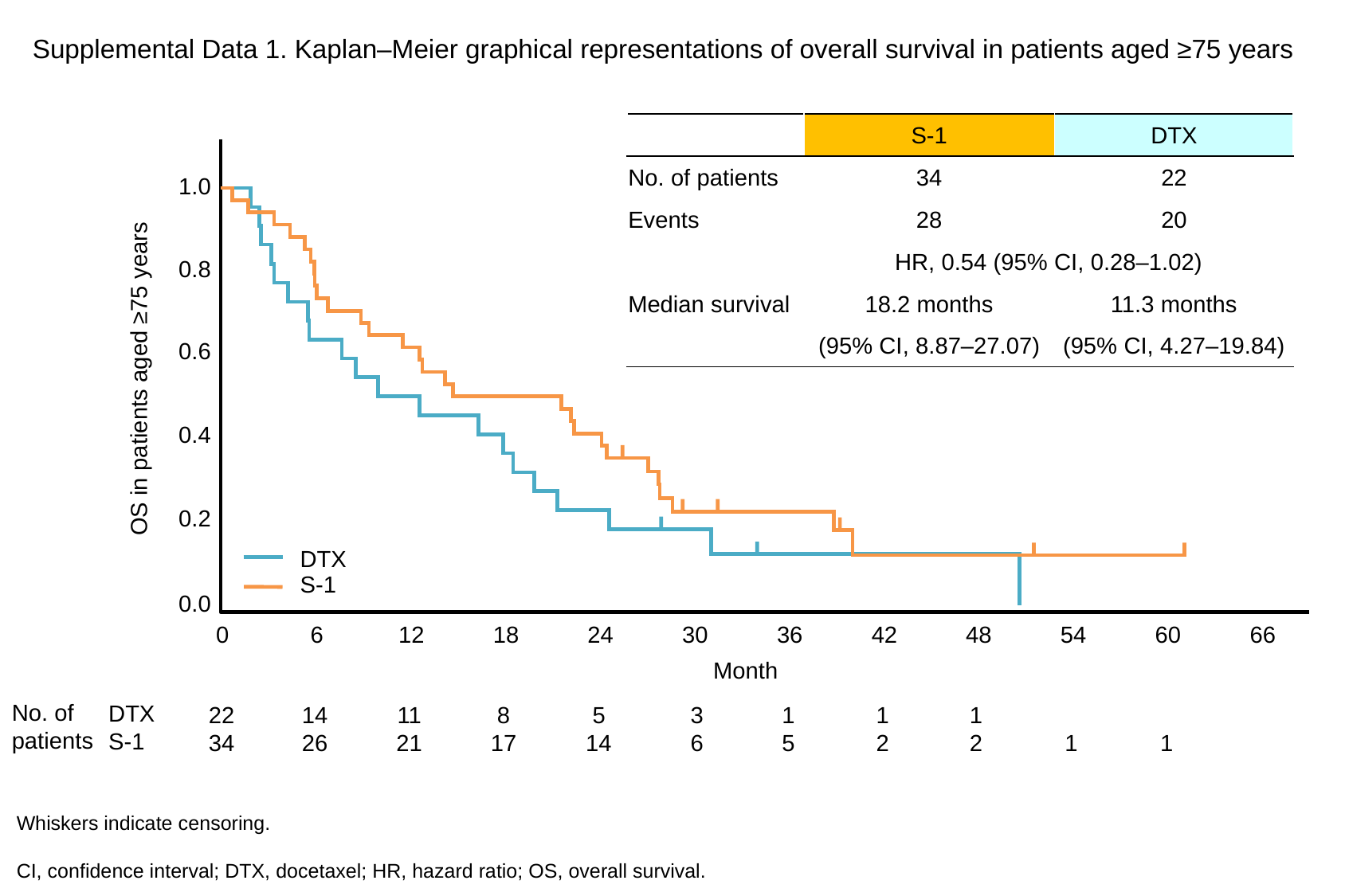

Supplemental Data 1. Kaplan–Meier graphical representations of overall survival in patients aged ≥75 years
| | S-1 | DTX |
| --- | --- | --- |
| No. of patients | 34 | 22 |
| Events | 28 | 20 |
| | HR, 0.54 (95% CI, 0.28–1.02) | |
| Median survival | 18.2 months | 11.3 months |
| | (95% CI, 8.87–27.07) | (95% CI, 4.27–19.84) |
1.0
0.8
0.6
0.4
0.2
0.0
OS in patients aged ≥75 years
DTX
S-1
0
6
12
18
24
30
36
42
48
54
60
66
Month
No. of patients
DTX
S-1
22
34
14
26
11
21
8
17
5
14
3
6
1
5
1
2
1
2
1
1
Whiskers indicate censoring.
CI, confidence interval; DTX, docetaxel; HR, hazard ratio; OS, overall survival.
