# Supplementary material for: Efficacy and Safety of S-1 Compared With Docetaxel in Elderly Patients With Advanced NSCLC Previously Treated With Platinum-Based Chemotherapy: A Subgroup Analysis of the EAST-LC Trial
Source: JTO Clin Res Rep. 2021 Jan 7;2(3):100142. doi: 10.1016/j.jtocrr.2021.100142 (PMC8474214; doi:10.1016/j.jtocrr.2021.100142)
Supplement: Supplemental Data 2 [file mmc2.docx]

**Supplemental Data 2.** Response rates assessed according to the Response Evaluation Criteria in Solid Tumors (version 1.1) (patients from the FAS with measurable lesions)

| **Response, n (%)** | **S-1 (N = 70)** | **DTX (N = 86)** |
| --- | --- | --- |
| CR | 0 (0) | 0 (0) |
| PR | 9 (12.9) | 12 (14.0) |
| SD | 27 (38.6) | 27 (31.4) |
| PD | 23 (32.9) | 32 (37.2) |
| NE | 11 (15.7) | 15 (17.4) |
| ORR (CR+PR) | 9 (12.9) | 12 (14.0) |
| DCR (CR+PR+SD) | 36 (51.4) | 39 (45.3) |

CR, complete response; DCR, disease control rate; DTX, docetaxel; FAS, full analysis set; NE, not evaluated; ORR, overall response rate; PD, progressive disease; PR, partial response; SD, stable disease.
