# Supplementary material for: Efficacy and Safety of S-1 Compared With Docetaxel in Elderly Patients With Advanced NSCLC Previously Treated With Platinum-Based Chemotherapy: A Subgroup Analysis of the EAST-LC Trial
Source: JTO Clin Res Rep. 2021 Jan 7;2(3):100142. doi: 10.1016/j.jtocrr.2021.100142 (PMC8474214; doi:10.1016/j.jtocrr.2021.100142)
Supplement: Supplemental Data 3 [file mmc3.docx]

**Supplemental Data 3.** Post-trial treatment^a^ (FAS)

| **Patients, n (%)** | **S-1 (N = 90)** | **DTX (N = 99)** |
| --- | --- | --- |
| No post-trial treatment | 31 (34.4) | 31 (31.3) |
| At least one post-trial treatment | 59 (65.6) | 68 (68.7) |
| Chemotherapy (including bevacizumab or cetuximab) | 54 (60.0) | 57 (57.6) |
| EGFR-TKI | 21 (23.3) | 24 (24.2) |
| ALK inhibitors | 2 (2.2) | 1 (1.0) |
| Investigational drug | 5 (5.6) | 10 (10.1) |
| Radiotherapy | 2 (2.2) | 0 (0) |
| Other | 1 (1.1) | 0 (0) |

^a^Patients may have received multiple post-trial treatments.

ALK, anaplastic lymphoma kinase; DTX, docetaxel; EGFR-TKI, epidermal growth factor receptor-tyrosine kinase inhibitor; FAS, full analysis set.
